# Use of Platelet Rich Fibrin (PRF)-Based Autologous Membranes for Tooth Extraction in Patients under Bisphosphonate Therapy: A Case Report

**DOI:** 10.3390/biomedicines7040089

**Published:** 2019-11-10

**Authors:** Alberto Pispero, Ivan Bancora, Antonious Khalil, Dario Scarnò, Elena M. Varoni

**Affiliations:** Dipartimento di Scienze Biomediche, Chirurgiche ed Odontoiatriche, University of Milan, Via Beldiletto 1, 20142 Milano, Italy; pispero.alberto@gmail.com (A.P.); ivanbancora@hotmail.it (I.B.); antonios.khalil@gmail.com (A.K.); darioscarno@yahoo.it (D.S.)

**Keywords:** oral medicine, oral surgery, bisphosphonates, platelet-rich fibrin, osteonecrosis

## Abstract

Tooth extraction in patients treated with bisphosphonates (BPs) for osteoporosis or cancer exposes the patient to the risk of osteonecrosis of the jaw. An autologous membrane using platelet-rich fibrin (PRF) is an innovative technique to promote wound healing, which allows obtaining a hermetic closure of the post-extractive surgical site without the need of mucoperiosteal flaps or periosteal releasing incisions. Here, we report the case of a 70-year-old woman, in therapy with alendronate for 12 years, requiring the upper right premolar extraction because of a crown fracture. After the tooth extraction performed under antiseptic and antibiotic coverage, the PRF autologous membrane was placed on the surgical wound to close completely the post-extraction site. Follow-up visits were carried out after one, two, four weeks and two months from the intervention. The complete re-epithelization of the wound was observed without signs of infection. The use of PRF for the closure of post-extraction sockets in patients taking BPs appears to be a promising alternative to the more invasive surgical procedures. Future clinical trials will be pivotal in elucidating the effectiveness of PRF to prevent BP-related osteonecrosis after tooth extraction.

## 1. Introduction

Bisphosphonates (BPs) and denosumab are bone antiresorptive agents, commonly prescribed for treating disorders of bone metabolism [1,2,3]. BPs at high dosage are useful in managing cancer metastases and other malignant diseases, including multiple myeloma; they are also prescribed at low dosage for the treatment of systemic conditions such as osteoporosis and Paget’s disease. These drugs have different mechanisms of action to reduce bone resorption via blockage of osteoclast differentiation: BPs act at an osteoclast level, while denosumab inhibits RANK-L.

A strong correlation between the use of these drugs and the risk of osteonecrosis of the jaws has been well documented in the literature, especially after a local trauma, mainly as tooth extraction [4,5]. This complication is characterized by an intraoral persistent area of bone exposure and/or signs of infection for a minimum period of 8 weeks that occurs in patients who are under current or previous treatment with BPs or other anti-resorbing drugs, without a clinical history of the head/neck radiotherapy [4,6,7]. 

Since the number of patients who have taken or are taking BPs is increasing in dental practice, a preventive approach, before starting BPs or denosumab therapies, is strongly recommended, in order to eliminate preliminarily the hopeless teeth and to treat dental foci of infection, thus avoiding the need of tooth extraction once the therapy has been commenced [8]. Rarely, the osteonecrosis has been associated with a local trauma due to the presence of incongruous removable prostheses, bone exostosis or to the insertion of dental implants [9]. The risk of developing osteonecrosis is related to the route of drug administration and dosage. More than 90% of cases reported in the literature are related to the intravenous use of zoledronic or pamidronic acid, usually prescribed for treating cancers affecting bone tissue. Zoledronate, in particular, is most frequently agent associated with osteonecrosis of the jaw, with a risk of 1% in the first year of intake to reach 21% after 3 years from the first administration. Patients taking other types of BPs, such as alendronate per os and at lower dosage for osteoporosis, however, are not exempted from the risk. 

The administration of systemic antibiotics in association with oral antiseptics in the pre- and/or post-surgical phases, the execution of atraumatic extraction procedures and the mobilization of mucoperiosteal flaps for primary closure of the surgical site are standard procedures of safety to prevent osteonecrosis in BP patients requiring oral surgery [3,9]. Neither international consensus nor the duration of the administration is available for which types of antibiotics should be used. Several protocols have been proposed, in particular using broad-spectrum antibiotics such as amoxicillin (1 g, 3 times/day) with or without clavulanic acid, also in combination with metronidazole (500 mg, 2 times/day), in case of allergy to penicillin, erythromycin (600 mg, 3 times/day), clindamycin (600 mg, 3 times/day) or ciprofloxacin (500 mg, 2 times/day). Lodi et al. proposed a protocol based on a combined antibiotic and antiseptic therapy that starts 3 days before surgery and continues for at least a week, and could be prolonged according to progress of the wound healing [9,10]. Intra-operative primary wound closure is, in this protocol, recommended to reduce bone infection [9,10], thus requiring mucoperiosteal flaps or periosteal releasing incisions and a more invasive procedure.

To date, biomaterial-based alternatives are pivotal in closing the surgical site with minimal invasiveness. In the present case report, we have investigated the use of platelet-rich fibrin (PRF) for this purpose. PRF, developed by Joseph Choukroun for uses in oral and maxillofacial surgery [11,12,13], is currently the most commonly studied platelet concentrate to facilitate the healing of post-extraction sockets. The preparation of PRF does not require the use of anticoagulants (bovine thrombin), as occurs for other platelet concentrates, such as platelet-rich plasma (PRP) and growth factors-rich plasma (PRGF). PRF is strictly autologous, producing negligible minimal immunological reaction and containing a mixture of patient-derived platelets, leukocytes, growth factors and cytokines. Two of the main advantages are related to the ease in manufacturing and the presence of bioactive elements. PRF requires, indeed, a single-step manufacturing procedure, where the autologous blood sample necessitates minimum blood manipulation [14]. PRF can be produced following fast and simple steps for collection and preparation, and patient’s blood can be drawn via a vacutainer before, during or after the surgical intervention. From a clinical point of view, PRF shows excellent handling properties, including resistance to traction and elasticity, and it can be easily sutured in a surgically selected position, even in combination with bone grafts [14,15]. Regarding bioactive elements, a plethora of growth factors and cytokines can be released from PRF constantly and steadily in the long term [15]. PRF includes transforming growth factor-beta 1 (TGF-β1), platelet-derived growth factor-BB (PDGF), insulin-like growth factor-1 (IGF-1) and vascular endothelial growth factors (VEGFs), as well as interleukin (IL)-1β, IL-4 and IL-6 [14]. The resulting three-dimensional PRF-based membrane/scaffold promotes tissue regeneration, and it can also act as functional support for the bone morphogenetic proteins (BMPs), from which osteoconductive properties can be further derived [9,10,11,12,16].

Here, we reported the successful case of a patient requiring dental extraction of a hopeless fractured tooth, who received BP therapy based on alendronate for 12 years. The surgical procedure, under antiseptic and antibiotic coverage, was performed by placing an autologous PRF membrane to close the post-extraction site.

## 2. Description of the Case

The patient, a 70-year-old woman with glaucoma and osteoporosis, came to our observation due to the suspected fracture of upper right second premolar (1.5 teeth). In her clinical history, she reported previous and current therapies with alendronate for 12 years (70 mg tablets, to be taken once a week). 

**Figure 1:**
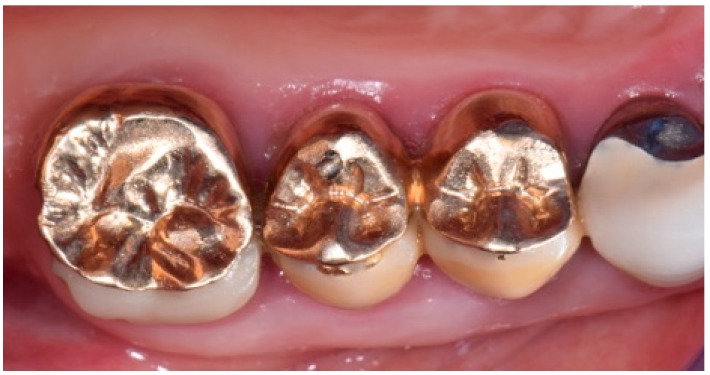
Initial intra-oral clinical view of the suspected fracture of upper right premolar (1.5 teeth) (quadrant I) of a patient, showing the premolars and molar (1.4, 1.5 and 1.6 teeth) prosthetically restored with gold-ceramic crowns.

The intra-oral examination showed the upper right premolars and molars (1.4, 1.5 and 1.6 teeth) prosthetically restored with gold ceramic crowns (Figure 1). 

**Figure 2:**
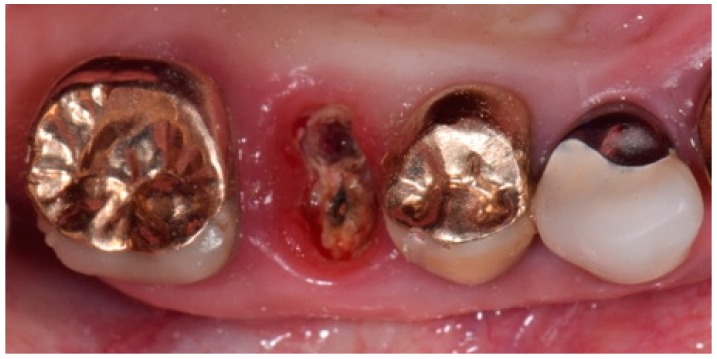
Intra-oral clinical picture after the crown removal, showing the fracture of upper right second premolar (1.5 teeth).

After gold ceramic crown removal, the fracture of upper right second premolar (1.5 teeth) was clinically visible (Figure 2). The tooth appeared hopeless, and thus the dental extraction was planned under the patient’s agreement.

Following the preventive protocol proposed by Lodi et al. to reduce the risk of BP-related osteonecrosis of the jaw, the patient underwent a professional oral hygiene session a week before the extraction, also starting a local antiseptic therapy with 0.2% chlorhexidine mouthrinse twice a day. The antibiotic therapy was set three days before the intervention (amoxicillin 1 g tablets: 1 tablet every 8 h) to be continued at least for one-week post-extraction [9].

**Figure 3:**
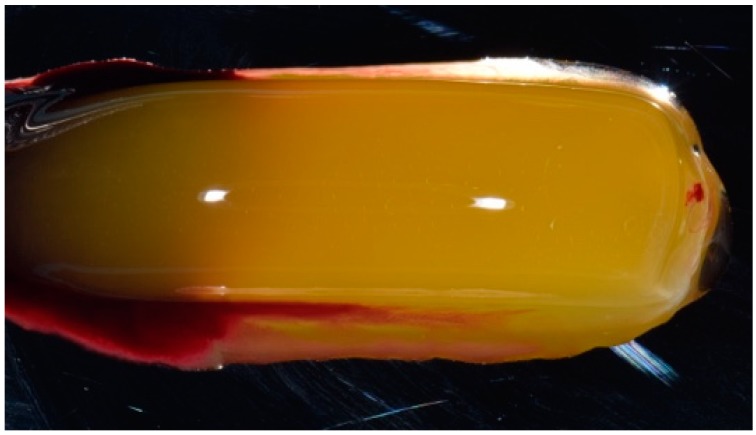
Platelet-rich fibrin (PRF) obtained from the patient’s blood sample.

During the day of the intervention, a blood sample (20 mL) was drawn from the patient and collected in two sterile tubes, without any type of anticoagulant. The PRF protocol was performed according to the Choukroun procedure [17] and the European Directive 2004/23/CE of 31 March 2004. The sample was centrifuged once at 3000 rpm for 10 min. At the end of the centrifugation, three distinct fractions were obtained: at the bottom of the tube, the concentrated red cells could be observed, while, at the top, there was the formation of the so-called platelet-poor plasma. The intermediate part was, instead, the dense PRF clot, which can be then clinically used in the form of an autologous membrane (Figure 3). 

**Figure 4:**
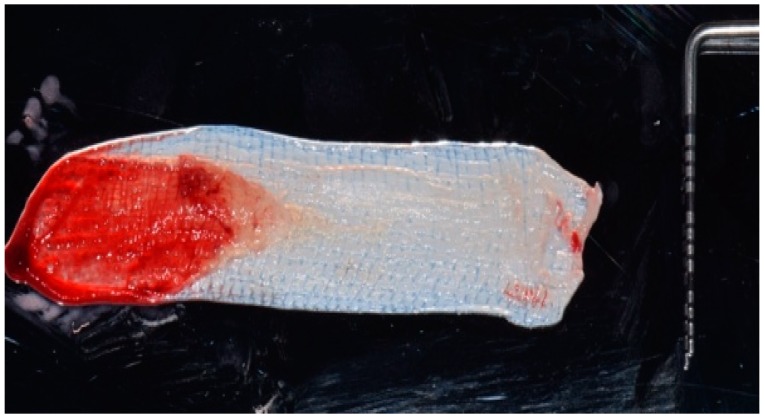
PRF membrane obtained from the patient’s blood sample.

After intra-oral local anesthesia via tissue infiltration (2% mepivacaine mg/ml + 1:100,000 adrenalin), the atraumatic extraction of the residual root of 1.5 tooth was performed. After irrigation with a sterile saline solution of the surgical site, the revision of the post-extraction socket was performed and the resulting exposed alveolar bone was covered with two layers of PRF (Figure 4).

**Figure 5:**
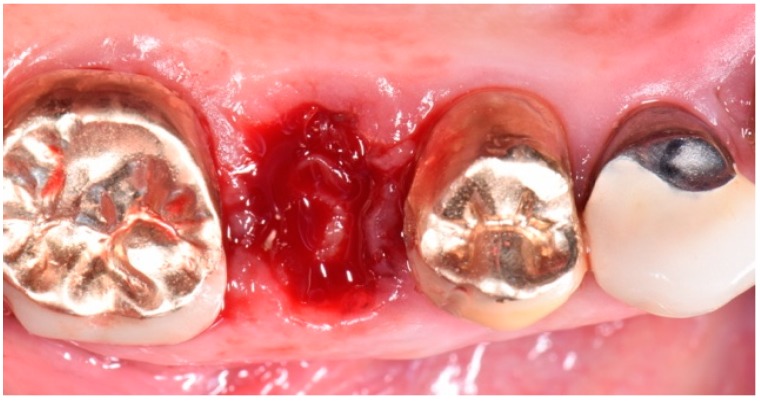
Insertion of the first layer of PRF membrane inside the post-extraction socket.

**Figure 6:**
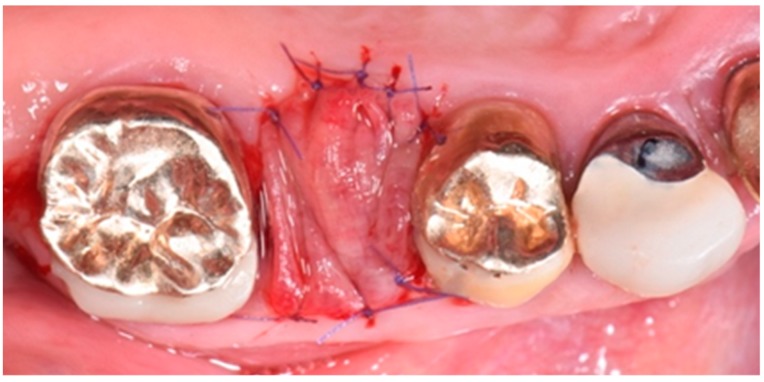
Placement of the second layer of PRF membrane over the first layer, in order to cover the post-extraction alveolar socket. The edges of the membrane were placed under the mucosal flaps and sutured to the surrounding gingiva.

The first layer of the PRF membrane was placed within an alveolar socket (Figure 5), while the second layer was positioned above the alveolar socket, with the margins located under the surgical flap and a resorbable 6.0 suture (Figure 6) was placed for stabilization. The complete wound closure of the post-extraction alveolar socket was obtained (Figure 5 and Figure 6).

The antibiotic regime was continued for 14 days after surgery, combined with application of 1% chlorhexidine gel three times a day at the surgical site. 

**Figure 7:**
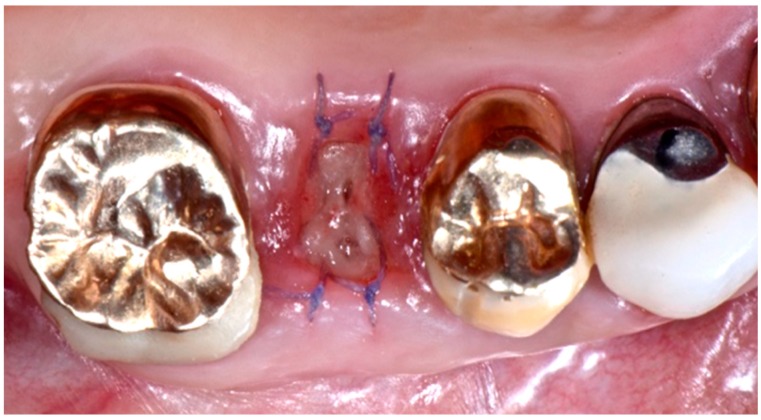
Intra-oral clinical view after 1 week from the intervention. Partial re-epithelization can be observed over the alveolar socket.

At a post-operative one-week follow-up, no signs of infection or inflammation were visible and there was new gingival tissue formation, which partially covered the surgical site (Figure 7).

**Figure 8:**
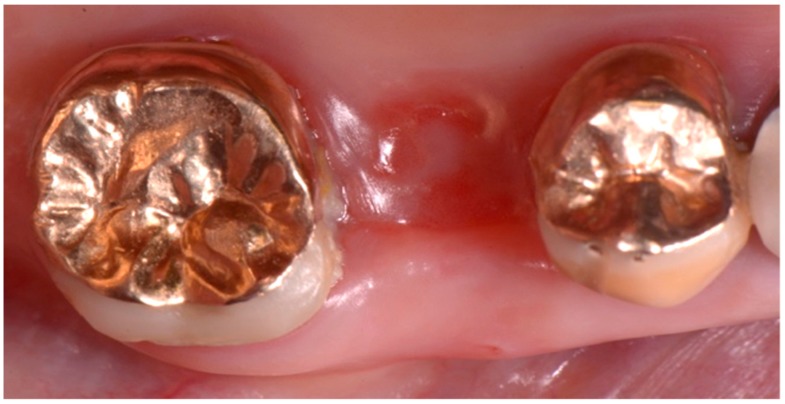
Intra-oral clinical view after 2 weeks from the intervention, showing that complete re-epithelization was visible at the surgical post-extraction site.

The residual sutures were removed at the end of the second post-operative week, when the complete re-epithelization of the surgical site was achieved, with newly regenerated mucosa (Figure 8).

**Figure 9:**
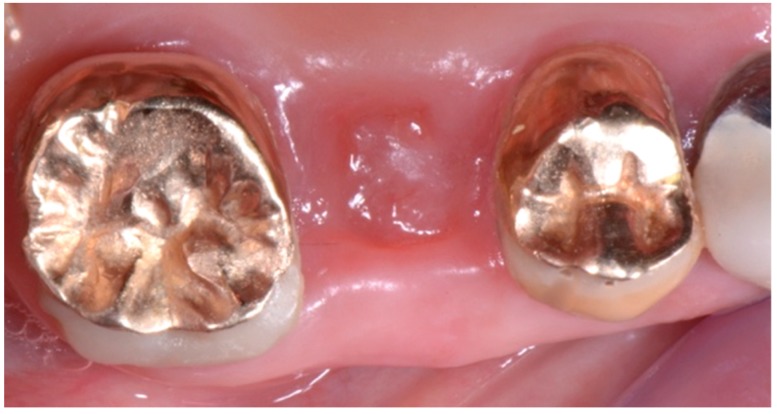
Complete re-epithelization of the surgical site after 2 months from the intervention.

No signs of inflammation, infection or exposed bone were detectable at a post-operative follow-up visit after 2 months (Figure 9). 

## 3. Discussion and Conclusions

BP drugs, usually prescribed for osteoporosis, hypercalcemia or bone metastases when the risk of fractures and pain are severe and could affect patient’s quality of life, have the aim of inhibiting osteoclast activity and consequently reduce the bone remodeling. After oral surgery, the BP mechanism of action hinders the bone wound healing related to the traumatism caused, for instance by a dental extraction. BPs are responsible of nearly 60% of osteonecrosis of the jaws [3], which in most of cases is triggered by dentoalveolar surgery. Many authors described clinical and pharmacological protocols to minimize the risk of developing osteonecrosis [1,4,5,8,9].

The benefits of using PRF autologous membranes, in the healing of post-extraction sites and improvement of post-operative pain, have been documented in the recent years [18,19]. PRF can stimulate the healing process both in terms of bone tissue regeneration by promoting osteogenesis within the post-extraction site and in terms of soft tissue regeneration by promoting re-epithelization [17,18]. Furthermore, suturing the PRF membrane over the extraction socket achieves a complete closure of the wound, as recommended in case of tooth extraction in BP patients to minimize bacterial contamination [9]. The latter can be achieved without the need of performing mucoperiosteal flaps or releases of the periosteum that could increase the patient’s post-operative morbidity. The use of PRF, thus, reduces invasiveness of the surgical procedure, which is limited to the atraumatic extraction of the tooth. 

Within the limitations of this case report (one patient receiving a single tooth extraction, who assumed BPs per os for osteoporosis), the use of PRF membrane, as an adjuvant for soft and hard tissue healing, appears promising to achieve the complete closure of post-extraction sockets with mini-invasiveness. Moreover, the handling properties of PRF membrane, which was easy to be placed and used, were clinically excellent, showing resistance and adaptability to the surgical wound and elasticity, such as providing surgeons with a suitable and simple positioning during the intervention [20,21]. PRF can be proposed as a valid alternative to traditional surgical extraction techniques that involve mucoperiosteal flaps and periosteal releases, in order to obtain a primary closure of the surgical wound. Further studies involving a larger sample of patients with different clinical pictures (different types of BP, dosage and route of administration; multiple tooth extractions with or without signs of infection) will be necessary to evaluate the success rate and the predictability of this technique in preventing BP-related osteonecrosis of the jaw after tooth extraction.
